# Reduced expression of cytochrome oxidases largely explains cAMP inhibition of aerobic growth in *Shewanella oneidensis*

**DOI:** 10.1038/srep24449

**Published:** 2016-04-14

**Authors:** Jianhua Yin, Qiu Meng, Huihui Fu, Haichun Gao

**Affiliations:** 1 Institute of Microbiology and College of Life Sciences, Zhejiang University, Hangzhou, Zhejiang, 310058, China; 2 Key Laboratory for Agro-Microbial Research and Utilization, Zhejiang Province Hangzhou, Zhejiang, 310058, China

## Abstract

Inhibition of bacterial growth under aerobic conditions by elevated levels of cyclic adenosine 3′,5′-*m*ono*p*hosphate (cAMP), first revealed more than 50 years ago, was attributed to accumulation of toxic methylglyoxal (MG). Here, we report a Crp-dependent mechanism rather than MG accumulation that accounts for the phenotype in *Shewanella oneidensis*, an emerging research model for the bacterial physiology. We show that a similar phenotype can be obtained by removing CpdA, a cAMP phosphodiesterase that appears more effective than its *Escherichia coli* counterpart. Although production of heme *c* and cytochromes *c* is correlated well with cAMP levels, neither is sufficient for the retarded growth. Quantities of overall cytochromes *c* increased substantially in the presence of elevated cAMP, a phenomenon resembling cells respiring on non-oxygen electron acceptors. In contrast, transcription of Crp-dependent genes encoding both cytochromes *bd* and *cbb*_3_ oxidases is substantially repressed under the same condition. Overall, our results suggest that cAMP of elevated levels drives cells into a low-energetic status, under which aerobic respiration is inhibited.

Among living organisms, prokaryotes thrive in every potential habitat on the Earth suitable for life because of their unparallel metabolic diversity. In many bacteria, central to regulation of metabolism is the cAMP (cyclic adenosine 3′,5′-*m*ono*p*hosphate)-Crp (*c*AMP *r*eceptor *p*rotein) regulatory system, as clearly illustrated in many bacteria, *Escherichia coli* in particular^1^,^2^. The primary role of the canonical cAMP-Crp system, revealed mostly by early studies on *Escherichia coli*, is to regulate uptake of preferred carbon sources and repression of genes required for utilization of less preferred ones, a process called carbon catabolite repression (CCR)^3^. However, this turns to be only the tip of the iceberg as more and more biological processes are reported to be regulated by the system in diverse bacteria^1^. By using a robust top-down physiological approach, You *et al.* recently demonstrated that the physiological function of the cAMP-Crp system is to coordinate the allocation of proteomic resources with different metabolic demands in different nutrient environments^4^. Although environmental cues that modulate cAMP signals vary depending on species or even strains (for example, several α-ketoacids in *E. coli*), there is a possibility that most, if not all, of relevant bacteria use the cAMP-Crp system the same way that *E. coli* does. That is, the transcription of Crp-dependent genes could be differently regulated by altered cAMP levels in response to environmental changes.

*Shewanella*, a genus of Gram-negative γ-proteobacteria thriving in diverse environments, possess highly adaptable metabolism, a quality that could be exploited for potential applications in bioremediation of heavy metals and energy generation via fuel cells^5^,^6^. While this subject has been a focus for more than two decades, the genus is now emerging as an important research model for general bacterial physiology. Many physiological traits displayed by shewanellae, mostly based on studies of the genus representative *Shewanella oneidensis*, are distinct, not found in *Escherichia coli* and other well-characterized model microorganisms. In addition, shewanellae are regarded as a reservoir for antibiotic resistance and the number of *Shewanella* species identified as pathogenic to animals including human being has been increasing with time^7^,^8^.

*S. oneidensis* is a strictly respiratory organism because the gene encoding 6-phosphofructokinase (PFK), an essential enzyme of glycolysis, is missing^9^. Moreover, the ability of *S. oneidensis* to utilize five- and six-carbon carbohydrates is rather poor because of the scarcity of enzymes for such sugars and their transport^10^. Despite this, the bacterium, probably all of shewanellae, is regarded respiratory versatile because it derives energy by coupling organic matter oxidation to the respiration of an array of terminal electron acceptors (EAs), such as oxygen, fumarate, nitrate, and metal oxides^5^. To date, how this bacterium adopts different metabolic modes in response to the availability of different EAs has been intensively studied, and some progresses have been made. First, Fnr (*f*umarate/*n*itrate *r*egulator), whose *E. coli* courterpart is the major player in respiration, has no significant role in bacterial physiology^11^. Second, *S. oneidensis* uses the Arc (*a*erobic *r*espiration *c*ontrol) system for regulating aerobic respiration without affecting genes in the tricarboxylic acid (TCA) cycle^12^. Third, it is evident that Crp is crucial in respiration because *crp* mutants are defective in utilizing several EAs, including oxygen, Fe^3+^, Mn^4+^, nitrate, nitrite, fumarate, and dimethyl sulfoxide (DMSO)^13^,^14^,^15^,^16^,^17^,^18^,^19^.

During aerobiosis, the primary targets of the cAMP-Crp regulatory system are genes encoding terminal reductases, including those reducing oxygen but traditionally called as cytochrome oxidases^16^,^17^. Cytochrome oxidases generate energy by coupling the oxidation of a respiratory substrate such as a *c*-type cytochrome or quinol to the reduction of oxygen to water^20^. Like in most bacteria, there are multiple cytochrome oxidases in *S. oneidensis*, two cytochrome *c* oxidases (a *caa*_3_-type and a *cbb*_3_-type) and a *bd*-type quinol oxidase^17^. For respiration of oxygen, cytochrome *cbb*_3_ is the predominant system whereas cytochrome *caa*_3_ is not of significance^17^. Cytochrome *bd*, on the other hand, appears to mainly facilitate adaptation to a variety of stress conditions, especially nitrite, although it is able to support growth when cytochrome *cbb*_3_ is absent^16^,^21^.

Initially observed in *E. coli* and later in other bacteria, aerobic growth is impeded when cAMP is present at concentrations of 0.5 mM or higher with certain sugars as carbon sources^22^,^23^. This effect of cAMP is attributed to accumulation of methylglyoxal (MG), which is a toxic intermediate produced from dihydroxyacetone phosphate (DHAP) by MG synthase (MGS)^22^,^24^.

We have found by chance, in the course of studies on the cAMP-CRP regulation of genes for nitrate and nitrite reductases^15^, that cAMP at 2 mM also retarded aerobic growth in *S. oneidensis*. However, a gene encoding an *E. coli* MGS homologue is missing in the *S. oneidensis* genome^9^. Thus, possibilities for the growth defect associated with cAMP include i) another protein functioning as MGS if MG is responsible, and (ii) a different mechanism. In this report, we describe the investigation of the subject. Our results demonstrate that cAMP at elevated levels retards growth mainly by compromising transcription of Crp-dependent genes for both the cytochrome *cbb*_3_-type and *bd* oxygen oxidases.

## Methods

### Bacterial strains, plasmids and culture conditions

The bacterial strains and plasmids used in this study are listed in Table 1. Sequences of the primers used in this study are available upon request. All chemicals are from Sigma-Aldrich Co. unless otherwise noted. *E. coli* and *S. oneidensis* were grown aerobically in Lysogeny broth (LB, Difco, Detroit, MI) at 37 and 30 °C for genetic manipulation. When appropriate, the growth medium was supplemented with the following: 2,6-diaminopimelic acid (DAP), 0.3 mM; ampicillin, 50 μg/ml; kanamycin, 50 μg/ml; gentamycin, 15 μg/ml.

Growth of *S. oneidensis* strains under aerobic conditions was measured at 600 nm (OD_600_) in either LB or MS defined medium, which contains 30 mM lactate as electron donor used as previously described^25^. For aerobic growth, mid-log phase cultures were inoculated into fresh media to an OD_600_ of ∼0.02 and shaken at 200 rpm at 30 °C.

### In-frame mutant construction and complementation

In-frame deletion strains were constructed using the *att*-based fusion PCR method as described previously^26^. In brief, two fragments flanking the genes of interest were amplified by PCR, and then linked by a second round of PCR. The fused fragments were introduced into plasmid pHGM01 using the Gateway BP clonase II enzyme mix (Invitrogen) according to the manufacturer’s instruction. Vectors carrying mutational constructs in *E. coli* WM3064, were subsequently transferred into *S. oneidensis* via conjugation. Integration of the mutagenized constructs into the chromosome was selected by resistance to gentamycin and confirmed by PCR. These transconjugants were grown in LB broth in the absence of NaCl and plated on LB supplemented with 10% sucrose. Gentamycin-sensitive and sucrose-resistant colonies were screened by PCR for deletions of the target genes. Mutants were verified by sequencing the region containing the intended mutations.

Plasmids pHG101 were used for genetic complementation of the mutants^27^. Wild-type genes and their adjacent promoters, were generated by PCR and cloned into pHG101. For inducible gene expression, genes of interest generated by PCR were introduced into pHGE-P*tac* under the control IPTG-inducible promoter P_*tac*_^28^. After verification by sequencing, the vectors were transferred into the relevant strains via conjugation for complementation and/or expression.

### Chemical assays

Cultures of 3 ml grown to an OD_600_ of ∼0.2 were subjected to filtering through a 0.22 μm nylon membrane for separation of cells and cell-free filtrate. The filtrate was immediately for cAMP assay, which was performed by using a commercially available kit (cAMP direct immunoassay kit, BioVision, http://www.biovision.com/camp-direct-immunoassay-kit-colorimetric-2862.html) according to the manufacturer’s instructions. The external cAMP levels were used to estimate the cAMP excretion rate by multiplying the specific growth rate and normalizing to OD_600_ values as described elsewhere^4^. The relative cAMP excretion rate for each mutant strain was given by comparing to that of the wild-type, representing the relative internal cAMP level because it is proportional to the cAMP excretion rate^29^. Amounts of MG and heme *c* from cells were measured following the procedures described elsewhere^22^,^30^. Standard curves were made with commercial agents each time.

### Viability assay

*S. oneidensis* strains grown to an OD_600_ of ∼0.2 were incubated with 0.4 mM MG or 4 mM cAMP for half an hour, then adjusted to approximately 10^7^ CFUs/ml, and followed by 10-fold serial dilutions. Ten microliters of each dilution was spotted onto LB plates. For nitrite susceptibility assay, ten microliters of each dilution of the untreated was spotted onto LB plates containing 5 mM nitrite. The plates were incubated at 30 °C before being read.

### Cytochromes *cbb*_3_ activity assay

Visual analysis of *cbb*_3_ activity was done by staining colonies with the agents for the Nadi Assay. Nadi reactions were carried out by the addition of a-naphthol and N′,N′-dimethyl-p-phenylenediamine (DMPD) on LB agar plates^31^. Colonies were timed for formation of the indophenol blue.

### SDS-PAGE and heme-staining

Unless otherwise noted, mid-log phase cells were harvested, washed with phosphate buffered saline (PBS), resuspended in the same buffer, and sonicated. Protein concentrations of the cell lysates were determined by the bicinchoninic acid assay (Pierce Chemical). The cell lysates were resolved by SDS-PAGE using 12% polyacrylamide gels and stained with 3,3′,5,5′-tetramethylbenzidine (TMBZ) as described elsewhere^32^.

### Promoter activity assay

The activity of various promoters was assessed using a single-copy integrative *lacZ* reporter system as described previously^33^. A fragment containing the sequence upstream of each operon from −300 to +1 (relative to the translation start codon) was amplified and cloned into the reporter vector pHGEI01 and verified by sequencing, These plasmids were then transferred by conjugation into relevant *S. oneidensis* strains. Plasmid pHGEI01 containing promoters of interest integrates into the chromosome and the antibiotic marker is then removed by an established approach^16^,^33^. Cells grown to the mid-log phase were collected and β-galactosidase activity assays were performed with an assay kit as described previously^27^.

### Other analyses

Student’s *t* test was performed for pairwise comparisons. Values are presented as means +/− standard deviation (SD) in the relevant figures.

## Results

### Growth inhibition by cAMP is not due to accumulation of methylglyoxal in *S. oneidensis*

**Figure 1:**
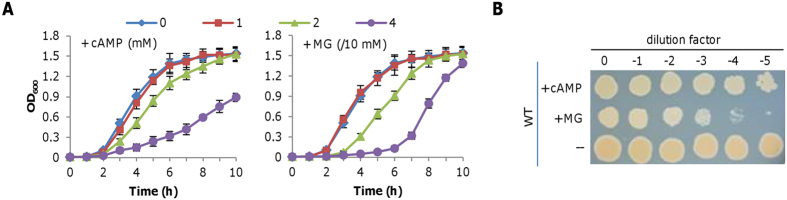
Effect of cAMP and MG on *S. oneidensis* physiology. (**A**) LB broth containing cAMP (0–4 mM) or MG (0–0.4 mM) was inoculated with mid-log phase *S. oneidensis* cultures (~0.2 of OD_600_), incubated (200 rpm) under aerobic conditions. (**B**) Viability assessment. Mid-log phase *S. oneidensis* cultures were incubated with 4 mM cAMP or 0.4 mM MG, serially diluted, and plated on LB plates. Photos were taken after 24 h. All experiments were performed at least three times with standard deviations presented as error bars in (**A**) and representative results presented in (**B**).

This investigation began with the chance observation that cAMP at 2 mM significantly retards aerobic growth of *S. oneidensis* in LB broth. To further assess the effect of cAMP on growth, we added cAMP of varying concentrations into liquid cultures (∼0.05 of OD_600_) prepared from the mid-log phase cells and monitored the consequences (Fig. 1A). While the addition of 1 mM cAMP hardly affected growth, the molecule at higher concentrations (2 and 4 mM) inhibited growth significantly and inhibition increased with cAMP levels. A similar trend was observed from the MS defined medium, but inhibition appeared more severe, with no visible growth in the presence of 4 mM cAMP (Fig. S1). Nevertheless, in both cases cell densities increased constantly when growth was not completely prohibited, a phenomenon not observed in *E. coli*, whose growth is completely arrested by much less cAMP (0.5 mM)^22^. In addition, we examined the effect of cAMP on viability. Cells of the mid-log phase (∼0.2 of OD_600_) were incubated with 4 mM cAMP for half an hour, properly diluted, and dropped on LB plates. As shown in Fig. 1B, cell viability of *S. oneidensis* appeared to be slightly reduced. But this was due to the growth defect because there was no difference in the number of viable cells between samples treated by cAMP and not from viable-cell counting (data not shown). Thus we concluded that cell viability is not affected significantly by cAMP, distinct from the fact that *E. coli* cells die rapidly because of the MG accumulation^22^. These contrasting phenotypes suggest that the growth defect of *S. oneidensis* resulting from exogenous cAMP may not be due to a toxic metabolite.

To rule out the possibility that MG underlies the growth defect in the presence of cAMP, we assessed impact of MG on growth. As shown in Fig. 1A, influences of cAMP and MG on growth were clearly different. We then examined MG on viability with cells prepared the same as above. Cells incubated with 0.4 mM MG for half an hour exhibited significantly reduced viability (Fig. 1B). Furthermore, despite of the lack of an *E. coli* MGS homologue, we examined levels of MG produced endogenously. In either rich or defined medium containing cAMP at concentrations that displays the strongest inhibition, MG was below the detection limit (data not shown). These data collectively conclude that the growth defect resulting from high concentrations of cAMP is not due to MG.

### An *S. oneidensis cpdA* mutant is defective in aerobic growth

**Figure 2:**
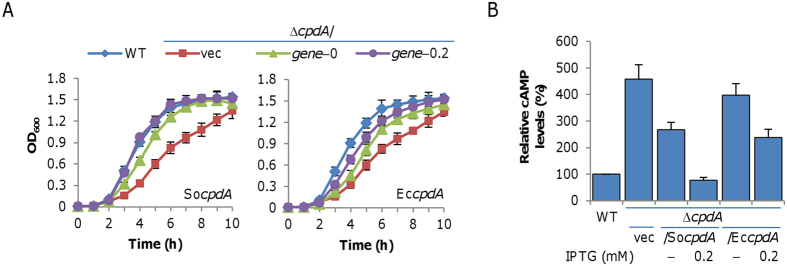
*S. oneidensis* CpdA is a cAMP phosphodiesterase. (**A**) Complementation of growth defect of the *cpdA* mutant in LB broth with the wild-type as control (WT). *S. oneidensis cpdA* (So*cpdA*) and *E. coli cpdA* (Ec*cpdA*) were placed behind the IPTG-inducible P_*tac*_ promoter as described in the experimental procedures. The *cpdA* mutants carrying empty vector (vec), *S. oneidensis cpdA* (So*cpdA*), and *E. coli cpdA* (Ec*cpdA*) were examined without IPTG or with 0.2 mM IPTG. (**B**) cAMP levels in cultures in (**A**) The averaged cAMP level in WT was set to 100%, to which cAMP levels in other strains were normalized. Experiments were performed at least three times with error bars representing the standard deviation.

Given the data presented above, we reasoned that mutants lacking enzymes that catalyze cAMP degradation are likely more sensitive to the molecule. To date, such enzymes for cAMP hydrolysis have not been characterized in *S. oneidensis*. However, CpdA (SO_3901) appears to be an *E. coli* cAMP phosphodiesterase homologue encoded in the *S. oneidensis* genome, with 45% identity in amino acid sequence and an E value of 2e-76 in a BLASTp analysis. To confirm that *S. oneidensis* CpdA functions as a cAMP phosphodiesterase, we constructed a *cpdA* in-frame deletion strain (Δ*cpdA*). It is immediately evident that growth of Δ*cpdA* was significantly impaired (Fig. 2A). To validate this phenotype, a copy of the *S. oneidensis cpdA* gene under the control of the IPTG-inducible promoter (P_*tac*_) was introduced into the mutant (Fig. 2A). Growth defect was partially corrected in the absence of IPTG because the promoter is slightly leaky^34^,^35^. With IPTG ranging from 0.01 to 0.2 mM, complementation was successful (Fig. 2A), indicating that the growth defect of the Δ*cpdA* strain was due to the intended mutation *per se*. More importantly, *E. coli cpdA* was also able to complement the growth defect, albeit not as effectively as its *S. oneidensis* counterpart (Fig. 2A).

To further provide evidence for the role of *S. oneidensis* CpdA as a cAMP phosphodiesterase, we assayed cAMP levels in relevant strains. Consistent with a previous report about an *E. coli* ∆*cpdA* strain^36^, intracellular levels of cAMP in an *S. oneidensis* ∆*cpdA* strain increased by over 2.5-fold (Fig. 2B). When either the *S. oneidensis* or *E. coli cpdA* gene was expressed, cAMP levels reduced greatly. Notably, with IPTG at 0.2 mM, cAMP levels between cells expressing the *S. oneidensis cpdA* and *E. coli cpdA* gene differed markedly, implying a difference in the efficacy of these two enzymes. Together with functional prediction based on the sequence, these data manifest that *S. oneidensis* CpdA functions to decompose cAMP.

### Growth inhibition by cAMP is dependent on CRP in *S. oneidensis*

**Figure 3:**
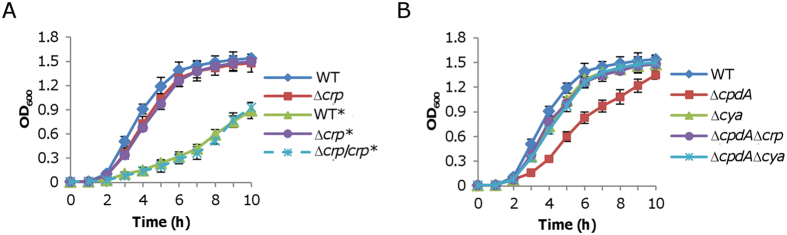
Growth inhibition by cAMP is dependent on Crp in *S. oneidensis*. (**A**) Growth of a *crp* mutant in the presence of 4 mM cAMP (marked with an asterisk). ∆*crp*/*crp* represents the *crp* mutant carrying a single copy of *crp* for complementation, which was successful^8^. (**B**) Growth of ∆*cpdA*, ∆*cya*, ∆*cpdA*∆*crp*, and ∆*cpdA*∆*cya* strains in LB broth. In both A and B, error bars representing standard deviations from at least three independent experiments.

To unravel the mechanism responsible for the cAMP inhibition in *S. oneidensis*, we first examined whether such effect of cAMP requires Crp. A *crp* deletion strain (∆*crp*), whose aerobic growth is only slightly impaired^11^, was subject to the analysis of cAMP effect. In contrast to the wild-type, the Δ*crp* strain was resistant to exogenous cAMP with respect to growth (Fig. 3A). As this observation was confirmed by genetic complementation with an integrative system described in our previous study^16^, it supports that Crp is essential for cAMP-induced growth deficiency.

In bacteria, cAMP is synthesized by adenylate cyclases (ACs)^3^,^36^. The *S. oneidensis* genome encodes three functional ACs, CyaA (SO_4312), CyaB (SO_3778) and CyaC (SO_1329), which have been characterized with respect to cAMP synthesis. Among them, CyaC is the major AC for cAMP production; the loss of all three ACs results in a phenotype similar to that of a *crp* mutant, in line with that both cAMP and Crp are essential to the physiological role of the cAMP-Crp complex^14^. To confirm that the growth defect requires the cAMP-Crp complex, we removed *crp* and *cya* (all three genes for ACs) from the Δ*cpdA* strain. In contrast to the Δ*cpdA* strain, the newly constructed Δ*cpdA*Δ*crp* and Δ*cpdA*Δ*cya* strains displayed normal growth, comparable to that of the Δ*crp* strain (Fig. 3B). Based on these results, we conclude that the growth defect, resulting from either addition of exogenous cAMP or the *cpdA* mutation, is dependent on the cAMP-Crp complex.

### Intracellular cAMP influences quantities of cytochromes *c*

**Figure 4:**
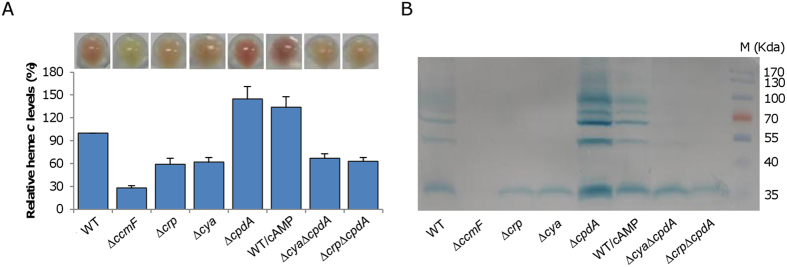
cAMP influences quantities of cytochromes *c*. (**A**) cAMP influences heme *c* levels. Mid-log phase cultures (~0.2 of OD_600_) of indicated strains were pelletted and photographed, then were lysed for quantition of heme *c* levels. The average amount of heme *c* from the wild-type strain was set to 100%. ∆*ccmF*, which could not produce cytochromes *c*, was used as negative control. WT/cAMP represents WT grown with 2 mM cAMP.(**B**) Proteins (10 μg per lane) extracted from the indicated samples were resolved by SDS-PAGE and analyzed by heme staining. All experiments were performed at least three times with standard deviations presented as error bars or similar results were obtained.

*S. oneidensis* colonies are brown-red on plates, largely because of more than 40 *c*-type cytochromes^11^,^37^,^38^. Previously, we reported that the loss of Crp decreases the levels of *c*-type cytochromes approximately by 60%^11^. During this investigation, we noticed that the color of Δ*cya* colonies (or cell pellets) was similar (Fig. 4A). In contrast, the color of Δ*cpdA* colonies was much deeper, so was the wild-type with 4 mM cAMP. We therefore hypothesized that the levels of *c*-type cytochromes increase with intracellular cAMP. To test this, heme *c* levels in relevant strains were determined with a Δ*ccmF* mutant used as negative control (Fig. 4A). The *ccmF* gene encodes a cytochrome *c* heme lyase, which is essential to *c*-type cytochrome maturation in *S. oneidensis*^28^,^39^. Compared to the wild-type, deletion of *cya* significantly lowered levels of heme *c*, which was comparable to that of the Δ*crp* strain and could be recovered by exogenous cAMP. In contrast, inactivation of *cpdA* elevated levels of heme *c*. Moreover, levels of heme *c* in the Δ*cpdA*Δ*crp* and Δ*cpdA*Δ*cya* strains were similar to those of the Δ*crp* and Δ*cya* strains. This observation was further confirmed by the profile of *c*-type cytochromes revealed by heme-staining (Fig. 4B). In a word, these data clearly show that the levels of *c*-type cytochromes in *S. oneidensis* increase with cAMP.

### cAMP-CRP regulates heme biosynthesis and cytochrome *c* maturation

**Figure 5:**
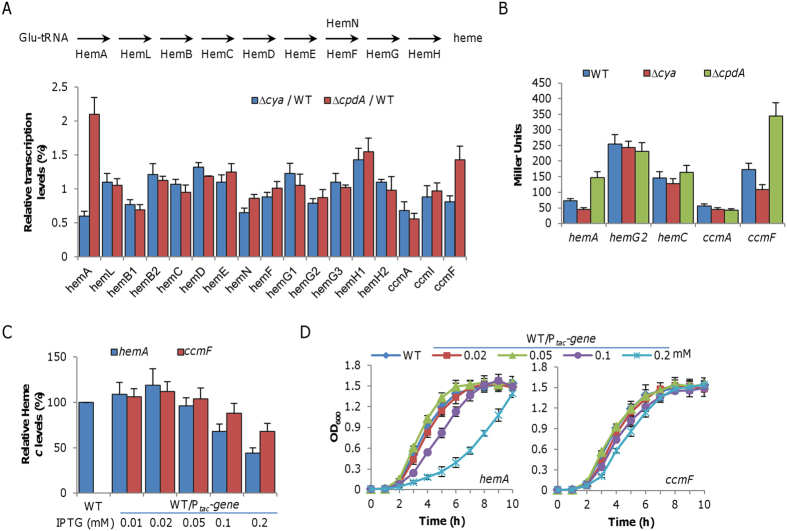
cAMP-CRP regulates heme biosynthesis and cytochrome *c* maturation. (**A**) Expression of the *hem* and *ccm* genes in ∆*cya* and ∆*cpdA* analyzed by qRT-PCR. Enzymes for heme biosynthesis are shown above: multiple candidates for HemB, HemG, and HemH are present; HemN and HemF HemN and HemF are coproporphyrinogen III oxidases, catalyzing the same reaction under different conditions. Cells of mid-log phase were prepared as described in the experimental procedures. The averaged expression level of each gene in mutants was compared to that in the wild-type. (**B**) Five genes were further analyzed by *lacZ*-reporter for confirmation. (**C)** Heme *c* levels in the wild-type overproducing HemA or CcmF. The average amount of heme *c* from the wild-type strain containing the empty vector was set to 100%. (**D)** Growth of the wild-type under indicated conditions as in C. All experiments were performed in triplicate and error bars indicate the standard error.

To elucidate the mechanism underlying growth defect and/or increased production of *c*-type cytochromes caused by the *cpdA* mutation, we focused on the heme synthetic pathway and the cytochrome *c* maturation system. *S. oneidensis* possesses the most common pathway for heme synthesis (Fig. 5A), as illustrated in *E. coli*, which entails nine reactions that converts glutamyl-tRNA to protoporphyrin IX^40^,^41^. Interestingly, there are multiple candidates for HemB, HemG, and HemH. To determine which of heme synthetic genes are affected by cAMP, we monitored abundance of the transcript of these *hem* genes by qRT-PCR provided that they are not organized into operons except *hemC* and *hemD*. In the ∆*cya* and ∆*cpdA* strains, the *hemA* gene was repressed and induced approximately 2-fold respectively, whereas the other *hem* genes were affected insignificantly (Fig. 5A). To confirm this observation, we used a *lacZ*-reporter to assay β-galactosidase activities driven by *hemA*, *hemG2*, and *hemC* promoters. Although robustness of these promoters differed substantially, they showed the same trend as observed from qRT-PCR (Fig. 5B).

HemA (glutamyl-tRNA reductase) catalyzes the first dedicated, rating-limiting step in heme synthesis^40^. To test whether HemA accounts for the phenotype of the ∆*cya* and ∆*cpdA* strains, we placed the *hemA* gene under the control of IPTG-inducible promoter P_*tac*_ to examine effects of HemA of varying quantities on heme *c* levels and growth of the wild-type. Surprisingly, HemA influenced the heme *c* levels in a dose-dependent way (Fig. 5C). With IPTG at no more than 0.05 mM, the heme *c* levels increased with HemA but further enhanced production of HemA by IPTG at 0.1 mM and above played an inhibitory role, resulting in significant reduction in heme *c* levels. Altered production of HemA also had an apparent impact on growth (Fig. 5D). When IPTG was added to levels more than 0.1 mM, growth was significantly retarded. In contrast, HemA induced by IPTG at 0.05 mM or lower did not exert any negative effect on growth.

We then examined whether the cytochrome *c* maturation system may be the cause for growth defect and increased heme *c* levels of the *cpdA* mutant. In contrast to the *hem* genes, the *ccm* genes are organized into three operons, *ccmABCDE*, *ccmI*, and *ccmFGH*^39^. qRT-PCR analysis of the transcript of the *ccmA*, *ccmI*, and *ccmF* genes revealed that the *ccmF* operon but not others was affected by the both *cya* and *cpdA* mutations (Fig. 5A). This observation was then confirmed by using the *lacZ*-reporter (Fig. 5B). Interestingly, forced production of CcmF by IPTG displayed an effect on heme *c* levels similar to that observed from HemA, although it appeared milder in the overproduction end (Fig. 5C). Consistently, growth was also similarly impacted (Fig. 5D). All together, these data suggest that HemA and CcmF, when present in certain range, can modestly affect quantities of *c*-type cytochromes, but in large excess exert a significant negative impact on *c*-type cytochrome production. Despite this, it is clear that neither of these two proteins appears to be critical for the growth defect of the *cpdA* mutant because their overproduction compromises quantities of *c*-type cytochromes.

### cAMP in excess inhibits activity of both cytochrome *bd* and *cbb*_3_ oxidases

**Figure 6:**
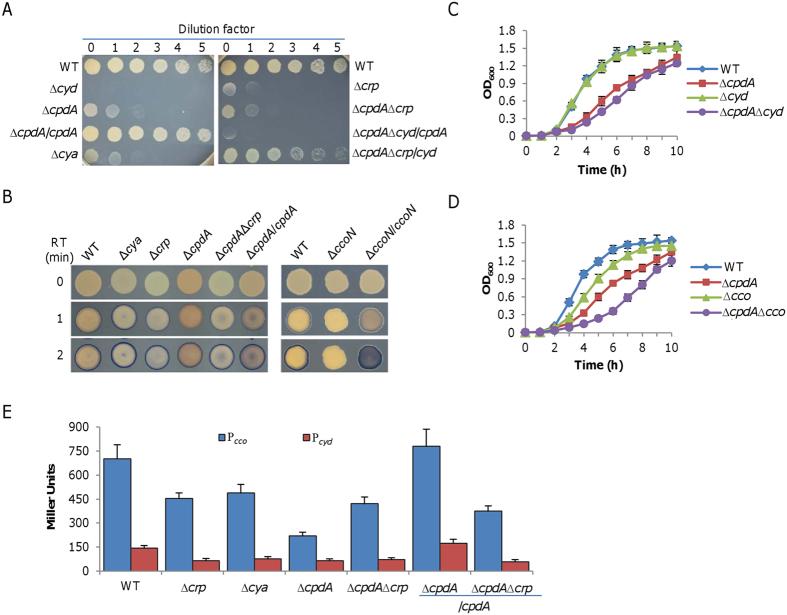
cAMP in excess inhibits cytochromes *cbb*_3_ and *bd*. (**A)** Effect of cAMP on susceptibility of indicated strains to 5 mM nitrite on LB plates. Cytochrome *bd* confers *S. oneidensis* resistance to nitrite. ∆*cpdA*/*cpdA*, ∆*cpdA*∆*crp*/*cpdA*, and ∆*cpdA*∆*crp*/*cyd* represent indicated mutants expressing *cpdA* or *cyd* by IPTG at levels sufficiently high for successful complementation as described in the text or previous publications. (**B**) Effect of cAMP on cytochrome *cbb*_3_ activity of indicated strains on LB plates by the Nadi assay. The method is based on the rapid formation of indophenol blue from colorless a-naphthol catalyzed by cytochrome *c* oxidase, using N′,N′-dimethyl-p-phenylenediamine monohydrochloride as an exogenous electron donor. Photos were taken at indicated times after the reaction started. The wild-type and Δ*ccoN* strains serve as positive and negative controls. (**C**) Growth of ∆*cpdA*∆*cyd* in LB. (**D)** Growth of ∆*cpdA*∆*cco* in LB. (**E**) Impacts of cAMP on expression of *cyd* and *cco* operons. Activities of the *cyd* and *cco* operon promoters from mid-log phase samples were assayed by a *lacZ*-reporter system used previously^17^. ∆*cpdA*/*cpdA*, and ∆*cpdA*∆*crp*/*cpdA* represent indicated mutants expressing *cpdA* by IPTG at levels sufficiently high for successful complementation as described in the text or previous publications. All experiments were performed at least three times with standard deviations presented as error bars or similar results were obtained.

To look further for answers addressing the growth defect of the *cpdA* mutant, we turned to cytochrome oxidases because these enzymes provide proton motive force for energy under aerobic conditions. Moreover, prior studies showed that the functional oxidases, *bd*-type (encoded by *cydABX*) and *cbb*_3_-type (encoded by *ccoNOPQ*), are under the direct control of the cAMP-Crp complex^16^,^17^,^21^. To test activities of the *bd*-type and *cbb*_3_-type oxidases in the Δ*cpdA* strain, we performed nitrite susceptibility assay and Nadi plate assay, respectively. Consistent with the notion that cytochrome *bd* confers resistance to nitrite in *S. oneidensis*^16^, a *cyd* null mutant (Δ*cyd*) was hypersensitive to nitrite (Fig. 6A). Like the Δ*crp* and Δ*cya* strains, the Δ*cpdA* strain displayed substantially increased susceptibility to nitrite, and loss of both *crp* and *cpdA* did not further elevate susceptibility. Importantly, this increased susceptibility due to the CpdA loss was restored to the level of wild-type by its expression *in trans*, only in the presence of cytochrome *bd*. Moreover, the phenotype was also complemented by forced production of cytochrome *bd*.

In the case of the cytochrome *cbb*_3_, similar results were obtained. With Nadi assay, which specifically detects cytochrome *c* oxidase-dependent respiration^31^, we visualized activities of the cytochrome *cbb*_3_ in relevant strains (Fig. 6B). As shown before^17^, loss of Crp compromised the cytochrome *cbb*_3_ activity. Surprisingly, the cytochrome *cbb*_3_ activity was most drastically reduced in the Δ*cpdA* strain, with the indophenol blue ring barely visible in one minute. This severe defect was dependent on Crp as the Δ*cpdA*Δ*crp* and Δ*crp* strains were indistinguishable.

Under standard conditions, the cytochrome *bd* is dispensable for aerobic growth of the *S. oneidensis* wild-type^17^. However, this was not observed with the *cpdA* mutation as the Δ*cpdA*Δ*cyd* strain had growth defect more severe than the Δ*cpdA* strain (Fig. 6C), suggesting that the cytochrome *bd* is crucial for supporting growth when the *cpdA* gene is absent. Similar results were obtained from the Δ*cpdA*Δ*ccoN* strain (Fig. 6D). Notably, the Δ*cpdA*Δ*cco* strain had the slowest growth rate when compared to the wild type, Δ*cpdA* and Δ*cpdA*Δ*cyd* strains, suggesting that in the Δ*cpdA* strain the cytochrome *cbb*_3_ still plays a predominant role in supporting growth as in the wild-type. These data, collectively, indicate that activities of both cytochrome *bd* and *cbb*_3_ oxygen reductases are impaired in the Δ*cpdA* strain, leading to growth deficiency.

To unravel the mechanism for reduced activities of both oxidases, we examined their expression levels in the Δ*cpdA* strain. As shown in Fig. 6E, there was no difference in activities of the *cyd* promoter in strains lacking any of tested genes, *crp*, *cya*, *cpdA*, or even two of them combined, suggesting that cAMP in absent and in excess has a similar regulatory effect on *cyd* expression. On the contrary, loss of *crp* or *cya* resulted in a modest reduction in *cco* expression but cAMP in overabundance was more detrimental. Altogether, these results suggest that the growth defect of the *cpdA* mutant is due to reduced production of both cytochrome *bd* and *cbb*_3_.

## Discussion

Cyclic nucleotides act as second messengers in diverse signaling cascades throughout all kingdoms of life, among which cAMP is first discovered and most extensively studied in bacteria^1^,^42^. The actions of cAMP are mediated by downstream cAMP-binding proteins, which are involved in diverse processes^43^,^44^,^45^,^46^. In bacteria, the central to cAMP-mediated regulation is formation of the cAMP-Crp complex, a transcriptional regulator of a number of metabolic operons, including those involved in the transport of substrates, glycolysis, the tricarboxylic acid cycle, and aerobic respiration^2^,^47^. It has been proposed that a key physiological role of the cAMP-Crp complex is to ensure the proteomic resources to be spent on distinct metabolic sectors as needed in different nutrient environments^4^. As a consequence, some carbon sources are transported and utilized when cAMP levels are manipulated. One of such examples is xylose, which can be converted to be DHAP, leading to production of toxic MG and thereby growth defect^22^. However, this is not the case in *S. oneidensis*. As our data presented here eliminate the possibility that MG is accountable for the growth phenotype in *S. oneidensis*, a different mechanism must exist.

The purpose of this study was to unravel the mechanism. The study was facilitated by an *S. oneidensis cpdA* mutant that stably maintains intracellular cAMP at levels sufficiently high to elicit a similar growth defect as the wild-type with 2 ~ 4 mM cAMP. *S. oneidensis* CpdA, A homolog of the *E. coli* counterpart, is verified to be a cAMP phosphodiesterase by cross-complementation. It should be noted that the *E. coli* CpdA could not fully complement the phenotype of the *S. oneidensis cpdA* mutant. With IPTG at 2 mM, forced production of *S. oneidensis* CpdA reduces the cAMP concentration below the wild-type level whereas cells producing *E. coli* CpdA exhibit only a 2-fold decrease in cAMP concentration (Fig. 2). This may not be surprising as the cAMP phosphodiesterase activity of *E. coli* CpdA is poor, with a rather high *K*_*m*_ for cAMP (~500 μM) relative to intracellular cAMP concentration^11^. Thus, at least in the context of *in vivo* data presented here, *S. oneidensis* CpdA functions more effectively than its *E. coli* counterpart.

As the growth defect of *S. oneidensis* caused by cAMP at elevated concentrations is dependent on Crp, we adopt the non-hypothesis-driven approach of testing whether some members of the cAMP-Crp regulon might be accountable for the defect when expressed differently. In addition to growth defect, the *cpdA* mutant, as well as strains lacking either ACs or Crp, differs from the wild-type in color of colony/pellet, which largely reflects the cellular amount of *c*-type cytochromes^11^. Apparently, cAMP levels correlate well with overall production of *c*-type cytochromes. Given that reduced quantities of *c*-type cytochromes, as due to loss of either ACs or Crp, do not significantly impede aerobic growth^11^,^13^,^14^, we tested whether *c*-type cytochromes in increased production could lead to retarded growth under aerobic conditions.

Amounts of *c*-type cytochromes are determined by two systems, responsible for the heme synthesis and cytochrome *c* maturation respectively. The heme synthesis is carried out by 10 enzymes (HemN and HemF for the same reaction under different conditions), of which only a few are found to be conditionally inducible^48^. In the present study, we found that *hemA*, whose product catalyzes the rating-limiting step^40^, is induced about 2-fold in the *cpdA* mutant. Previous studies have shown that HemA in *Salmonella typhimurium* responds to heme availability at the level of protein lifetime^49^,^50^: when heme is abundant, it binds to HemA to promote degradation of the latter. While whether the stabilization of *S. oneidensis* HemA is also an issue remains unknown, its induction by increased concentrations of cAMP, to our knowledge, is unprecedented. Seemingly, this regulation by cAMP-Crp is indirect because by prediction there is no Crp-binding site located upstream of the *hemA* gene^11^,^51^. However, given the negative effect of overproduced HemA on overall amounts of *c*-type cytochromes, the possibility that HemA plays an important role in the growth defect appears small. Intriguingly, this is also true of CcmF, the cytochrome *c* lyase. We therefore conclude that neither heme synthesis nor cytochrome *c* maturation is accountable for growth defect or increased level of *c*-type cytochromes observed from the *cpdA* mutant.

Rather, increased levels of heme *c* may be a result of concerted upregulation of many cytochrome *c* genes because more than two thirds of them are predicted to be under the direct control of the cAMP-Crp complex^11^,^13^,^14^,^33^,^51^. This surely gains support from heme-staining analysis (Fig. 4). We have previously shown that anaerobic respiration of various EAs favors overall cytochrome *c* production^11^,^14^, a scenario resembling the *cpdA* mutant to some extent. It has been suggested that cAMP concentrations increase in response to the low internal energetic status in *E. coli*, promoting catabolism and inhibiting anabolism^4^,^52^. In *S. oneidensis*, a similar notion has been proposed^17^. Thus, it seems logic that elevated cAMP drives cells into a low-energy mode, favoring respiration of non-oxygen EAs. As a consequence, genes encoding proteins important for respiration of oxygen are repressed, such as those for cytochrome *cbb*_3_ and *bd*. We propose that this explains the growth defect.

According to previous reports, *S. oneidensis* cAMP-Crp binds to DNA motifs similar to its *E. coli* counterpart whereas Crp alone fails in binding^11^,^15^,^16^,^17^,^18^,^19^. Data presented here reveal that cAMP at varying levels impacts expression of Crp-regulon members differently: the *cyd* operon (*bd*) behaves the same in cAMP-deficient and -overproduction strains whereas the *cco* operon (*cbb*_3_) is further repressed by increased concentrations of cAMP. This is consistent with the finding that *S. oneidensis* Crp functions in a dose-dependent manner^16^. Coincidently, a study of *E. coli* demonstrates that cAMP-binding has a biphasic effect on site-specific DNA-binding by Crp^53^.

In recent years, cAMP-Crp complexes with distinct features have been found. In *Mycobacterium*, Crp can not only operate at extremely high levels of cAMP, based on the finding that the intracellular cAMP levels are as high as 3–4 mM^54^, but also bind to DNA in a specific manner and regulate transcription without cAMP^55^. In *Pseudomonas putida*, a bacterium that also has an incomplete glycolysis pathway (lacking PFK), Crp exhibits an affinity binding of cAMP approximately 1000 times higher than that of *E. coli* Crp^56^,^57^. A consequence of these differences is that the Crp regulons of these bacteria, including *S. oneidensis*, differ drastically from that of *E. coli*, as suggested in *P. putida*^58^.

## Additional Information

**How to cite this article**: Yin, J. *et al.* Reduced expression of cytochrome oxidases largely explains cAMP inhibition of aerobic growth in *Shewanella oneidensis*. *Sci. Rep.* **6**, 24449; doi: 10.1038/srep24449 (2016).

## Supplementary Material

Supplementary Information

## Figures and Tables

**Table 1:** Strains and plasmids used in this study.

Strain or plasmid	Description	Source or reference
*E. coli* strain		
DH5α	Host strain for plasmids	Lab stock
WM3064	Donor strain for conjugation; Δ*dapA*	W. Metcalf, UIUC
*S. oneidensis* strain		
MR-1	Wild type	Lab stock
HG0266	Δ*ccmF* derived from MR-1	26
HG0624	Δ*crp* derived from MR-1	11
HG2364	Δ*ccoN* derived from MR-1	17
HG3901	Δ*cpdA* derived from MR-1	This study
HGCYA	Δ*cya* (Δ*cyaA*Δ*cyaB*Δ*cyaC*) derived from MR-1	This study
HGCCO	Δ*cco* (Δ*ccoNOPQ*) derived from MR-1	8
HGCYD	Δ*cyd* (Δ*cydABX*) derived from MR-1	21
HG3901-0266	Δ*cpdA*Δ*ccmF* derived from MR-1	This study
HG3901-0624	Δ*cpdA*Δ*crp* derived from MR-1	This study
HG3901-CYA	Δ*cpdA*Δ*cya* derived from MR-1	This study
HG3901-CCO	Δ*cpdA*Δ*cco* derived from MR-1	This study
HG3901-CYD	Δ*cpdA*Δ*cyd* derived from MR-1	This study
Plamid		
pHGM01	Ap^r^ Gm^r^ Cm^r^ suicide vector	26
pHGE-P*tac*	IPTG-inducible P*tac* expression vector	28
pHGEI01	Integrative *lacZ* reporter vector	33
pHGE-P*tac*-*cpdA*	Vector for expressing *cpdA*	This study
pHGE-P*tac*-*EccpdA*	Vector for expressing *E. coli cpdA*	This study
pHGE-P*tac*-*hemA*	Vector for expressing *hemA*	This study
pHGE-P*tac*-*ccmF*	Vector for expressing *ccmF*	This study
pHGE-P*tac*-*cyd*	Vector for expressing *cydABX*	8
pHGEI01-*cpdA*	Vector for measuring *cpdA* expression	This study
pHGEI01-*hemA*	Vector for measuring *hemA* expression	This study
pHGEI01-*ccmF*	Vector for measuring *ccmF* expression	This study
pHGEI01-*cco*	Vector for measuring *cco* expression	This study
pHGEI01-*cyd*	Vector for measuring *cyd* expression	This study
pHGEI01-*hemG2*	Vector for measuring *hemG2* expression	This study
pHGEI01-*hemC*	Vector for measuring *hemC* expression	This study
pHGEI01-*ccmA*	Vector for measuring *ccmA* expression	This study
