# Supplementary material for: Reduced expression of cytochrome oxidases largely explains cAMP inhibition of aerobic growth in Shewanella oneidensis
Source: Sci Rep. 2016 Apr 14;6:24449. doi: 10.1038/srep24449 (PMC4830989; doi:10.1038/srep24449)
Supplement: Supplementary Information [file srep24449-s1.pdf]

Supplemental materials of

Reduced expression of cytochrome oxidases largely explains cAMP inhibition of aerobic growth in *Shewanella oneidensis*

Jianhua Yin[a†], Qiu Meng[a†], Huihui Fu[a], and Haichun Gao[a,b]*

[a]Institute of Microbiology and College of Life Sciences, Zhejiang University, Hangzhou, Zhejiang, 310058, China

[b]Key Laboratory for Agro-Microbial Research and Utilization, Zhejiang Province Hangzhou, Zhejiang, 310058, China

[†]These authors contributed equally to this work.

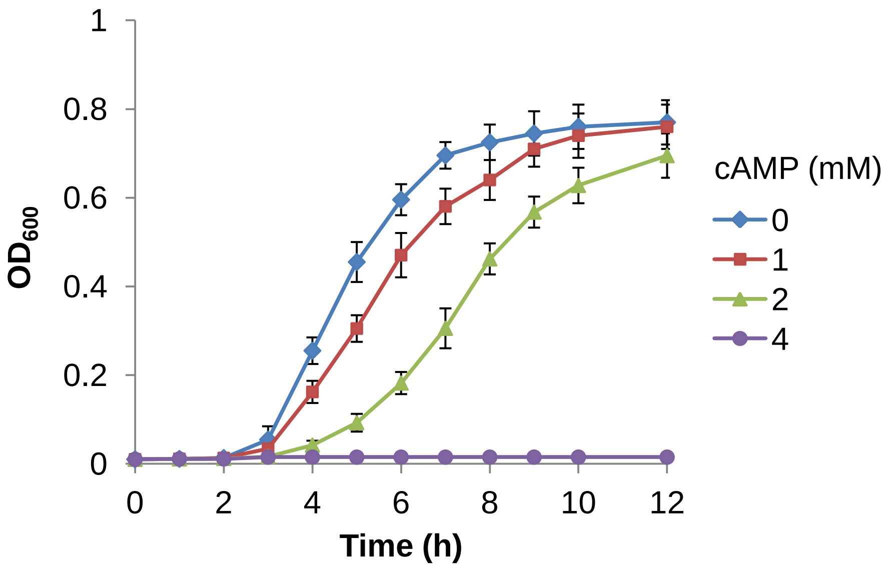

Figure S1. Effect of cAMP on *S. oneidensis* growth in defined medium. MS defined medium containing cAMP (0 – 4 mM) was inoculated with mid-log phase *S. oneidensis* cultures (~0.2 of OD_{600}), incubated (200 rpm) under aerobic conditions. All experiments were performed at least three times with standard deviations presented as error bars.
